# Castleman disease and SLE in a G6PD-deficient Marfan patient: a case report and literature review

**DOI:** 10.1186/s13317-020-00138-w

**Published:** 2020-11-03

**Authors:** Sami Alhoulaiby, Lina Okar, Haya Samaan, Hisham Qalaani

**Affiliations:** 1 Department of General Surgery, Faculty of Medicine, Damascus University, Damascus, Syria; 2 Department of Neurology, Faculty of Medicine, Damascus University, Damascus, Syria; 3 Department of Hematology, Internal Medicine, Faculty of Medicine, Damascus University, Damascus, Syria

**Keywords:** Marfan syndrome, SLE, Systemic lupus erythematosus, G6PD deficiency, Castleman disease

## Abstract

**Introduction:**

Marfan syndrome, G6PD deficiency, systemic lupus erythematosus (SLE), and Castleman disease are four distinctive, thoroughly investigated entities whose coincidence was never reported. However, occurrence in pairs was sporadically mentioned in literature.

**Case presentation:**

We report a 15-year-old Caucasian G6PD deficient Marfan male patient, who presented with tonic–clonic seizures, fever, a hemolytic episode, and general symptoms. After the discovery of hepatosplenomegaly, malar rash, and painless lymphadenopathy, further testing diagnosed a multifocal Castleman disease of the hyaline vascular subtype and systemic lupus erythematosus with lupus nephritis that got 35 points on the 2019 EULAR/ACR criteria. G6PD deficiency, SLE & Castleman disease, and seizures were handled medically with eventual improvement in the patient’s condition.

**Discussion and conclusion:**

It is extremely rare to discover the gathering of these four diseases in the same patient. Marfan syndrome and G6PD deficiency were proven by respective clinical and laboratory examinations. Castleman disease that tends to occur in older age groups was confirmed via pathological study of a lymph node biopsy, which was compatible with the HHV-8 negative type reported in Asian countries. SLE is part of the differential diagnosis for Castleman disease, yet the newest evidence strongly supports its presence as a distinct entity. However, no concrete proof is available to suggest a causative relationship between the four of them, rather than a coincidental occurrence.

## Introduction

Marfan syndrome (MFS) and G6PD deficiency are two well-known utterly investigated genetic entities that are mostly diagnosable in childhood. On the other hand, systemic lupus erythematosus (SLE) and Castleman’s disease (CD) are idiopathic diseases, whose multisystemic symptoms tend to be interchangeable and diagnostically challenging [1, 2], taking into account the ample range of differential diagnoses of lupus [3].

The simultaneous gathering of these four medical conditions has never been reported.

In this article, we report the first case of a 15-year-old Caucasian male with a history of Marfan and G6PD, who developed SLE, seizures, and Castleman disease.

## Case presentation

A 15-year-old Caucasian male was admitted to the neurology department after four tonic–clonic generalized seizures in the past ten days, accompanied by incontinence, worsening headache partially responsive to NSAIDs (Ibuprofen 600 mg), and one spike of fever, nausea, and vomiting with no photo- or phonophobia.

**Fig. 1:**
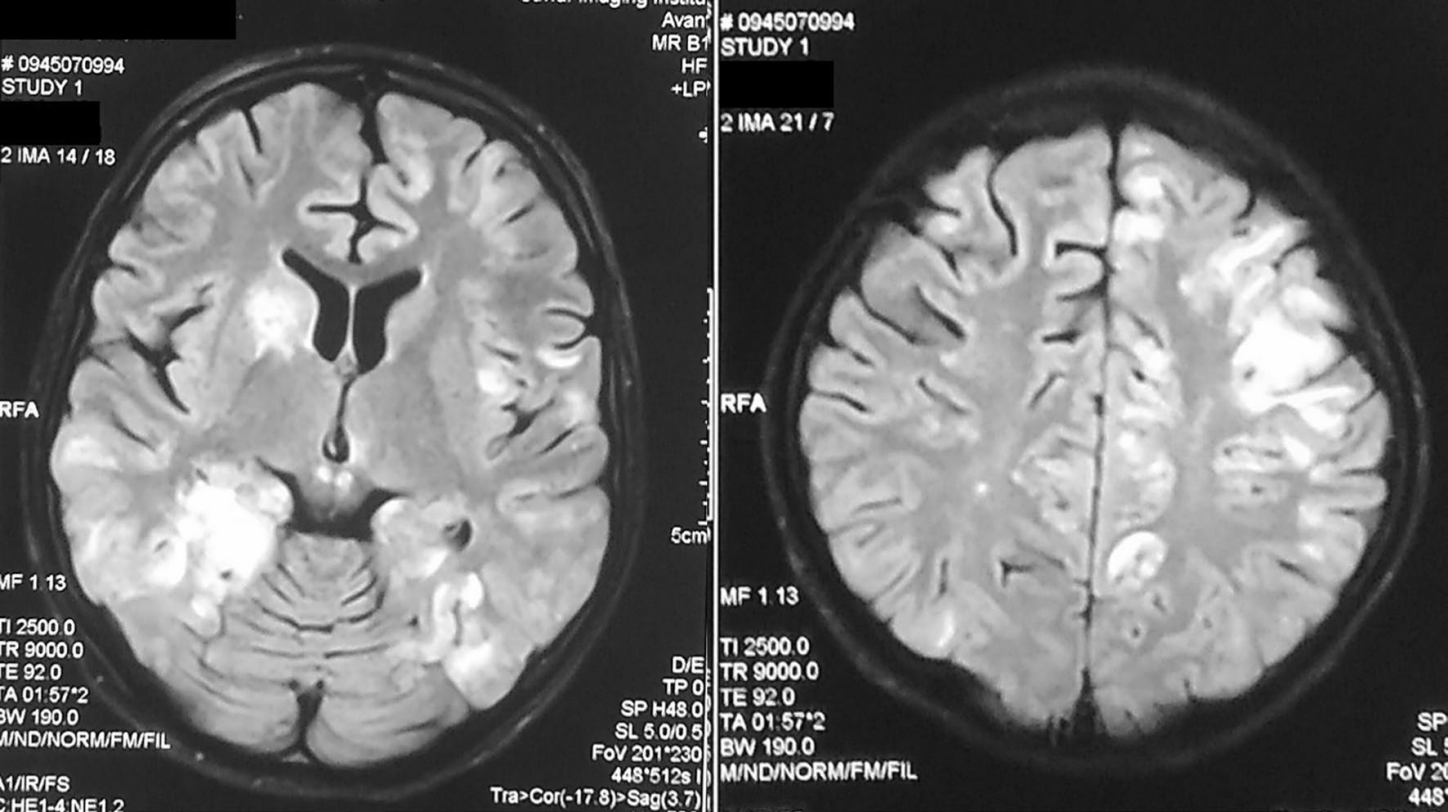
T2 MRI imaging presenting high signals in the cortical and subcortical area (right) and in the caudate nucleus and midbrain (left)

The patient’s history includes Marfan syndrome and G6PD deficiency with a familial background. A few days before admission, an outpatient brain MRI for the seizures was ordered, and it discovered the presence of multiple T2-high-intensity lesions in the cortical and subcortical level, the right caudate nucleus, midbrain, pons, medulla oblongata and cervical spinal cord that would be caused by acute diffuse encephalomyelitis (Fig. 1.). Upon this imaging, the patient was referred to our hospital for further investigation.

**Fig. 2:**
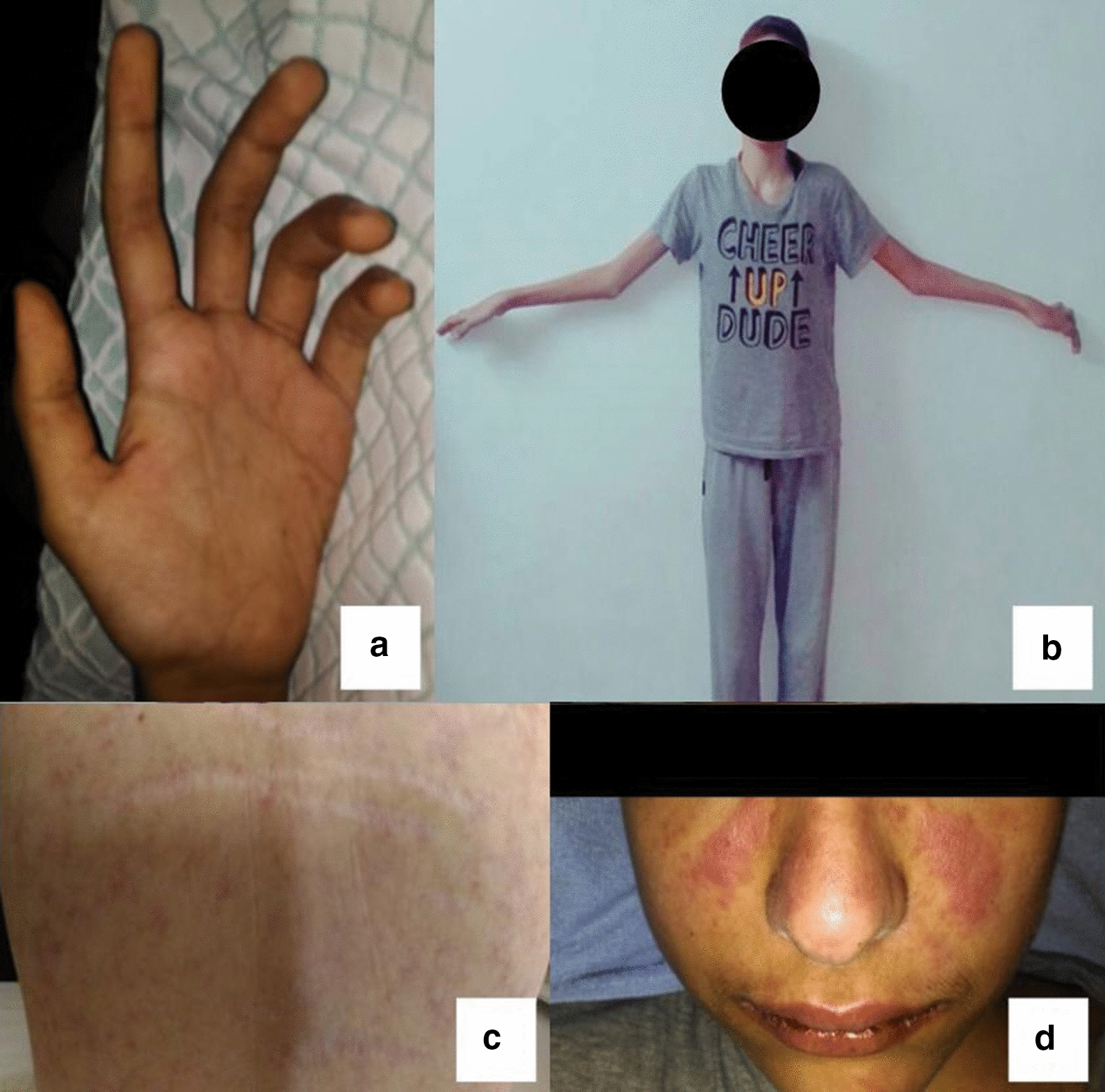
Marfan syndrome hallmarks: claw-shaped hand with long fingers (**a**). Marfanoid habitus with wide arm span (**b**). Striae with capillary dilation (**c**). SLE malar butterfly rash (**d**)

Physical examination on admission uncovered the following positive findings: first-degree exertional dyspnea, 38 °C oral temperature, BP: 210/110 mmHg, pulse: 130 bpm, neck stiffness, and positive Kernig and Brudzinski (both upper and lower) signs. Upper and lower extremities had a normal tone and diminished strength (3/5) and reflexes except for the left upper limb where intensified reflexes (+ 4) were examined. The hand was claw-shaped with long fingers, lucid lumbrical and hypothenar atrophy along with Marfan characteristic features (Fig. 2a–c). In addition, the head inspection detected a facial malar rash, erythematous spots on the palatine, and buccal mucosa and ulceration on the lower lip (Fig. 2d). In abdominal palpation, hepatomegaly 3 cm under the costal margin and splenomegaly also spanning 3 cm were felt on the respective midclavicular line, with painless lymphadenopathy. Otherwise, the rest of the examination was normal. Upon admission, laboratory tests revealed anemia with no other abnormality, and CSF analysis indicated the presence of a few RBCs and lymphocytes. Although the CSF protein levels were significantly high, glucose was only borderline low (Table 1)

**Table 1:** Abnormal laboratory tests on admission

Test	Result	Reference range
Blood analysis		
RBCs	3.6 * 10^6^/mm^3^	4.5–5.5 * 10^6^/mm^3^
Hemoglobin	10 g/dL	13–16 g/dL
Hct	31.2%	38–53%
MCV	86 fL	82–96 fL
MCH	25 pg	27.5–33.2 pg
Cerebral spinal fluid analysis		
WBCs	10 cells	Up to 5 cells
Lymphocytes	100%	
RBCs	10 cells	0 cells
Total protein	1432 mg/dL	100–400 mg/dL
Lactate	19.6 mg/dL	10–22 mg/dL
Glucose	41 mg/dL	40–70 mg/dL
ADA	11 U/L	Upto 9 U/L

*WBCs* white blood cells, *RBCs* red blood cells, *Hct* hematocrit, *MCV* mean corpuscular volume, *MCH* mean corpuscular hemoglobin, *ADA* adenosine deaminase

At first, the patient received empirical antibiotics for suspected meningitis in addition to levetiracetam for the seizures. After the blood pressure remained high, we added valsartan/hydrochlorothiazide (160 mg/12.5 g) combination. Follow-up broad laboratory tests including expansive iron testing indicated the continuance of anemia, high ESR (55 mm/1 h), a drop in total protein (5.5 g/dL Reference 6.2–8) and albumin (2 g/dL Reference 3.3–5.3) with normal globulins, an important low iron-binding capacity (TIBC) (163 µg/dL Reference: 250–425 µg/dL) plus a high ferritin (356 ng/dL Reference: 21–275 ng/dL), along with markedly low G6PD enzyme (0.11 U/g Reference: 7–20.5 U/g). Urine analysis was abnormal either as it showed positive RBCs, WBCs, cylinders, and protein (Table 2.) In order to exclude possible differential diagnoses for his unexplained fever, testing for common infections (Typhoid fever, Maltese fever, Hepatitis, and AIDS) was requested, nonetheless, the results of the respective tests (Widal, Wright, HBsAg, Anti HCV and HIV Ab/Ag) were completely negative. Later, the results for the CSF culture came back with total negativity for all possible causes of meningitis including fungi, herpes simplex (HSV) and tuberculosis, therefore, the empirical antibiotics were discontinued.

**Table 2:** Urine analysis

Test	Result	Reference range
pH	5	
Protein	+	
Hemoglobin	+ +	
Leucocytes	30 cell/mm^3^	Up to 10 cell/mm^3^
Erythrocytes	220 cell/mm^3^	Up to 10 cell/mm^3^
Cylinders	Granular +	

**Fig. 3:**
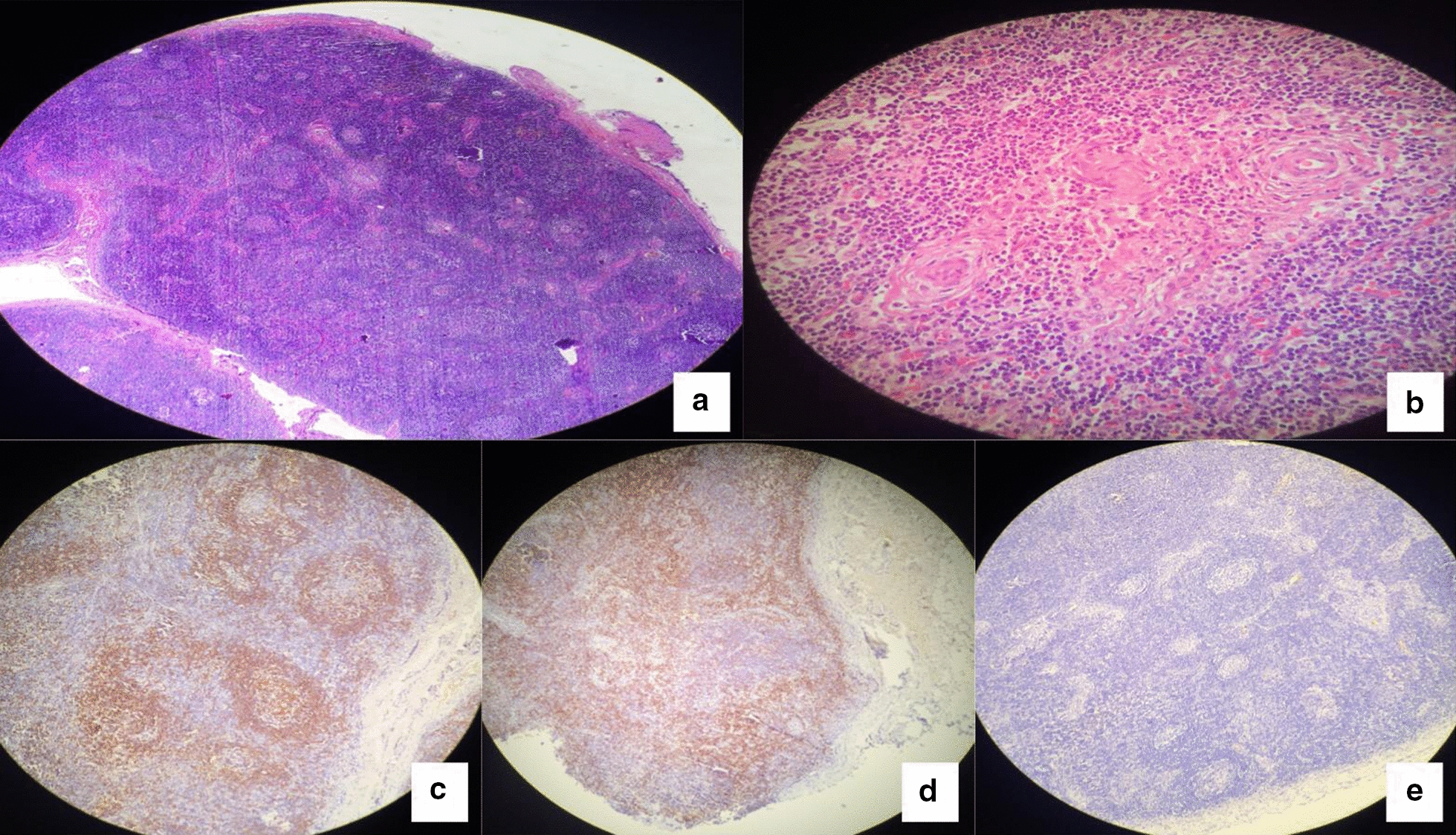
Lymph node biopsy presenting the general appearance of Castleman’s disease (**a**), Onion-skinning sign (**b**), Positive CD20 immunostaining for B-cells (**c**), Positive CD3 immunostaining for T-cells (**d**) and negative CD30 immunostaining (**e**)

The patient was then referred to all relevant departments for consultation and the following positive findings were documented: a tricuspid regurgitation, hepatosplenomegaly with normal portal vein, a slightly hyperechoic renal cortex and a moderate volume of fluid in the pelvis. A Lymph node biopsy described hyperplastic follicles with raws of lymphocytes around central hyalinization (onion-skinning sign) penetrated by hyalinized blood vessels, which was consistent with multifocal Castleman’s disease (MCD) of the hyaline vascular subtype (Fig. 3. A & B). Immunostains presented positivity for CD20 of the B cells, CD3 of the T cells, and CD23 with negative CD30, further confirming Castleman disease (Fig. 3. C, D & E).

For the following days, the values of WBCs, RBCs, and platelets kept on declining steadily (Table 3.). Immunological tests returned negative for both coombs tests, with low c3 protein [41.6 mg/dL Reference: 90–180 mg/dL], positive ANA IgG [1/320], positive anti-DNA antibodies at a dilution of 1/320, whilst lupus anticoagulant, antiphospholipid antibodies, anti ß2 glycoprotein I, anticardiolipin, p-ANCA and c-ANCA were negative altogether. A diagnosis of SLE was made combining the clinical manifestation and the laboratory results. Concurrently, the 24-h urine analysis uncovered a total protein of 4919 mg (normal up to 100 mg/24 h) with normal creatinine. This raised the suspicion of lupus nephritis, therefore, prednisolone 1 mg/kg was added to the patient’s medication and he was referred to undergo a kidney biopsy.

**Table 3:** Follow up on CBC values

Day	1	5	8	9	10	11	12	13	16
WBCs	7.2	4.5	3.8	3	2.8	2.5	2.4	2	1.5
Hgb	10	8	8.1	7.2	7.4	6.8	7	6.9	6.5
Platelets	170	128	130	148	148	144	125	119	100

**Fig. 4:**
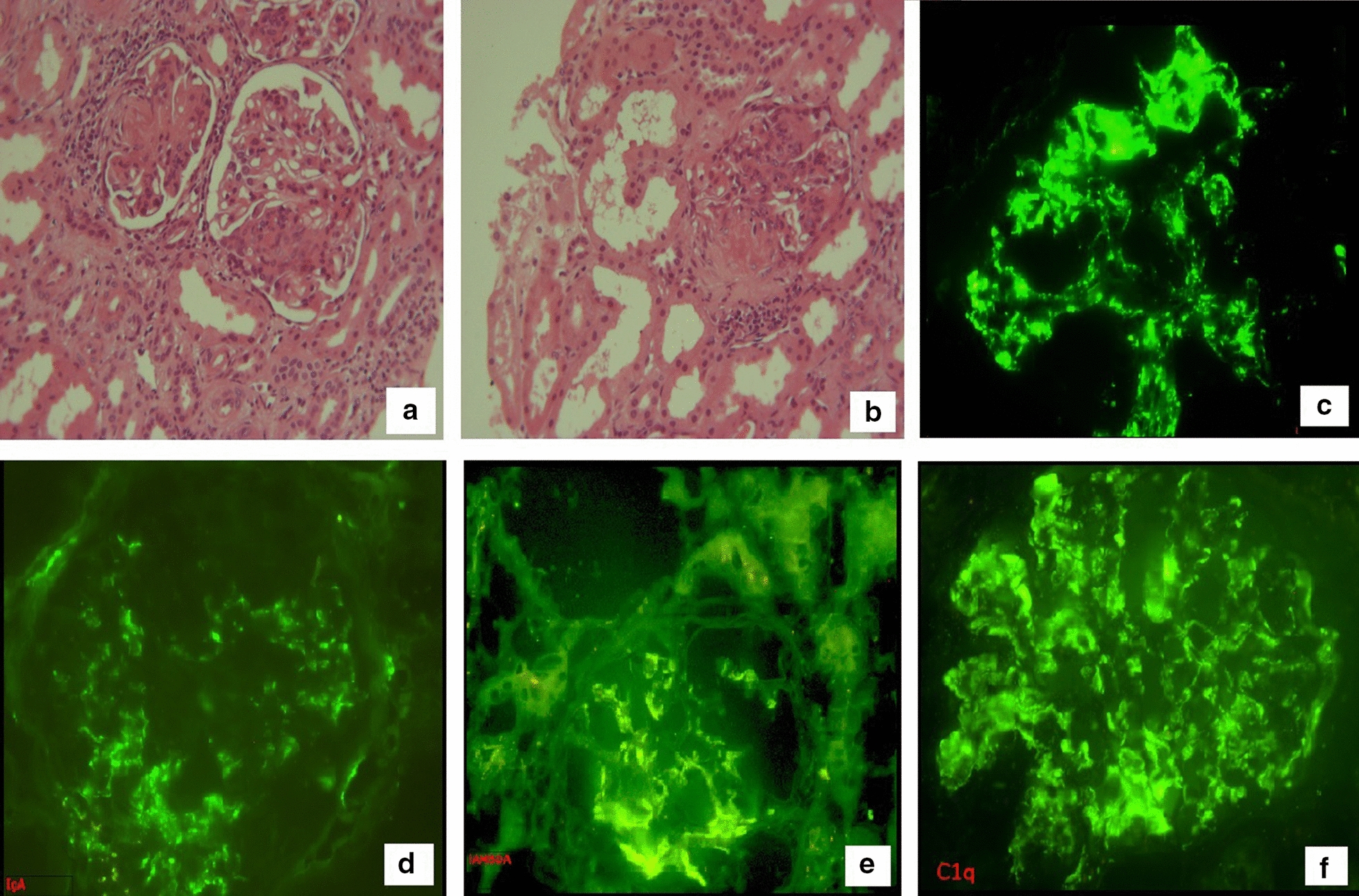
Kidney biopsy with immunofluorescence: capillary proliferation (**a**), adhesions to Bowman capsule, fibrinoid necrosis and karyorrhexis (**b**), positive IgG fluorescence (**c**), positive IgA fluorescence (**d**), positive lambda chains € and C1q (**f**)

Pathologic examination of the kidney biopsy (Fig. 4.) described segmental proliferative lesions consisting of endocapillary proliferation, adhesions to Bowman’s capsule, glomerular epithelial cell hyperplasia with fibrinoid necrosis and karyorrhexis. Some glomeruli showed segmental scars, while the others had thin and delicate glomerular basement membranes. The mesangial areas were expanded by mesangial PAS-positive matrix and moderate mesangial hypercellularity. Mason stain revealed interstitial fibrosis in 10% of the renal cortex. The tubules had a mild acute injury and the blood vessels were mildly fibrotic in the subintima (25% occlusion of the lumen). Lastly, Congo stain was negative for amyloid. Additionally, immunofluorescence detected positivity in the glomerular basement membranes and mesangium for IgG, Kappa, Lambda, C3, and C1q. The pathologist concluded the diagnosis as focal proliferative glomerulonephritis consistent with lupus nephritis (ISN/RPS 2003 classification, Class III).

To address the isolated weakness in the left upper limb, electromyography uncovered active hypoinnervation in the muscles innervated by the left ulnar nerve with a total absence of voluntary activity in the abductor digiti minimi muscle, and poor activation of first left interosseous and flexor carpi ulnaris muscles. Additionally, signs of general chronic decreased innervation in all of the four limbs were recorded. Nerve conduction study, however, revealed the absence of sensory potentials in the sural nerve, and by stimulating the left ulnar nerve no motor activity could be recorded in the left-hand muscles except for a minimal function in the flexor carpi ulnaris. The conclusion of these two tests was consistent with moderate chronic axonal sensorimotor polyneuropathy with increased intensity in the periphery of the lower limbs and an isolated severe axonal mononeuropathy in the left ulnar nerve consistent with hypoperfusion injury.

Tacrolimus (1 mg twice daily) was added to the regimen, and the patient was released from the hospital with close follow up on tacrolimus, prednisolone, and levetiracetam. The dose of tacrolimus needed multiple adjustments until it reached the therapeutic range with 3 mg morning and 2 mg evening doses. One month later, all signs of lupus had subsided.

## Discussion

The coincidence of CD, SLE, MFS, and G6PD deficiency has never been reported. This applies for the co-occurrence of Marfan with G6PD deficiency, and for G6PD deficiency with Castleman disease. A rare occurrence of SLE with MFS in a teenage girl was reported [4], and another case described familial occurrence in two sisters with accompanying rheumatoid arthritis [5]. MFS with CD is even more exceptional with only one reported case about pulmonary manifestations of Castleman’s disease presenting in a 50-year-old Marfan female [6]. Lupus with G6PD was found only in one article reporting 88 patients [7], and sporadic reports mentioned a scarce coexistence possibility of SLE with Castleman’s disease. [8–10].

Multifocal Castleman’s disease is a systemic disease with widely described manifestations that usually occurs in the 50–65 years of age group. It is rare in children, yet possible just like our 15-year-old patient. [11] Among many, it presents with lymphadenopathy, splenomegaly, and systemic inflammatory symptoms, however, the gold standard for diagnosis is the histopathologic findings. [12] Human herpesvirus 8 (HHV8) that accompanies Acquired Immune Deficiency Syndrome (AIDS) has a causative relationship with MCD in the United States, but the patients in Asia are usually HHV8 free [2]. As described earlier, our patient has a biopsy-proven MCD that was not caused by HHV8 due to the absence of HIV. The diagnosis of Marfan syndrome and G6PD deficiency was also based on clinical and laboratory investigations that were consistent with the patient’s clinical and familial history. SLE diagnosis was confirmed based on clinical and serological findings. Positive ANA and anti-ds-DNA antibodies, low C3 protein, lupus nephritis class III, acute cutaneous manifestations, seizures, and the evident fluid accumulation all sum up to 35 points on the 2019 EULAR/ACR criteria. [13].

And with such extreme proteinuria (around 5 g/24 h), the low serum albumin is totally understandable.

Castleman’s disease and SLE pose a diagnostic dilemma. Crossover symptoms can be frequently seen, and each disease criteria calls for the exclusion of all other possibilities including one another. Both diseases share presentations of fever, thrombocytopenia, proteinuria, and renal dysfunction. ~ 30% of MCD cases have positive autoantibodies like ANA, and 15–30% of SLE have histopathology similar to MCD. [2, 13–15] The positive anti dsDNA is highly specific for SLE, and both diseases meet their respective major criteria and most of the minor criteria as well. Three reports mentioned above consider the two diseases as distinctly present, not one mimicking another. A decision to specify the major disease of them is impossible with our current knowledge, and such intertwined symptoms.

The presenting complaint was the seizures. CSF analysis with its elevated lymphocytes and protein, and low glucose with no detectable infectious organisms suggests an inflammatory origin, which is compatible with the diffuse opacities seen on the MRI. The occurrence of abnormal CSF values with positive signs (nuchal rigidity, Brudzinski, and Kernig signs) but with negative organisms might be explained by an SLE related aseptic meningitis. Seizures are known to accompany SLE in 5–15% of cases as established by two reviews [16, 17], while they rarely accompany CD, relating chiefly to a focal cerebral localization [18], and appearing to be only coincidental with Marfan and G6PD deficiency [19–21]. The focal localization of seizures in CD instead of generalization, prioritizes SLE as the primary cause. We cannot deny the minuscule possibility of seizures happening in the context of the connective tissue disturbance (i.e. Marfan) as reported by Chu NS, but while the reported Marfan patients with epilepsy had angioid streaks in the retina or coloboma of the iris, our patient had a normal eye examination [20].

With these four thoroughly investigated and proven entities, this becomes the first case in the literature to describe their coincidence.

There is no evidence of a causal relationship between the four diseases, which is most probably coincidental.

The suitable treatment for each of the diseases was administered and led to the improvement of the patient’s symptoms and general status.

## Conclusion

This article presents the first of a kind display of mainly unrelated diseases. The following complication with seizures—which could be due to several factors—is interesting. Such a sophisticated case responded to the individual treatments: anticonvulsants, corticosteroid, and immunotherapy, which is worthy to highlight.

## Data Availability

All relevant data is incorporated into the production of this article.

## Abbreviations

MFS: Marfan syndrome
SLE: Systemic lupus erythematosus
G6PD: Glucose-6-phosphate dehydrogenase
CD: Castleman disease
mg: Milligram
BP: Blood pressure
bpm: Beats per minute
